# Differential analysis of high-throughput quantitative genetic interaction data

**DOI:** 10.1186/gb-2012-13-12-r123

**Published:** 2012-12-26

**Authors:** Gordon J Bean, Trey Ideker

**Affiliations:** 1 Bioinformatics and Systems Biology Program, University of California, San Diego, 9500 Gilman Drive, Dept. 0419, La Jolla, CA 92093-0419, USA; 2 Department of Bioengineering, University of California, San Diego, 9500 Gilman Drive MC 0412, La Jolla, CA 92093-0412, USA; 3 Institute for Genomic Medicine, University of California, San Diego, 9500 Gilman Drive, 0642, La Jolla, CA 92093, USA; 4 Department of Medicine, University of California, San Diego, 9500 Gilman Drive, # 0671, La Jolla, CA 92093-0671, USA

## Abstract

Synthetic genetic arrays have been very effective at measuring genetic interactions in yeast in a high-throughput manner and recently have been expanded to measure quantitative changes in interaction, termed 'differential interactions', across multiple conditions. Here, we present a strategy that leverages statistical information from the experimental design to produce a novel, quantitative differential interaction score, which performs favorably compared to previous differential scores. We also discuss the added utility of differential genetic-similarity in differential network analysis. Our approach is preferred for differential network analysis, and our implementation, written in MATLAB, can be found at http://chianti.ucsd.edu/~gbean/compute_differential_scores.m.

## Background

Genetic interactions are functional dependencies between genes, which become apparent when the phenotypic effect of one mutation is altered by the presence of a second. In model organisms such as yeast, genetic interactions can be rapidly assessed through the systematic construction of double mutants and measurement of quantitative phenotypes such as growth rate. Quantitative interactions may be positive or negative, indicating less or more severe double mutant phenotypes than expected from the single mutant phenotypes. Many large genetic network maps have been constructed from high-throughput genetic interaction screens in yeast, providing insight into the global landscape of interactions within the cell as well as the functional relationships between specific components of biological processes and pathways [1-5].

Recently, we used genetic interaction mapping in a 'differential mode' to compare the changes in genetic networks across experimental conditions [6-8]. To demonstrate this approach, called differential epistasis mapping, we compared the difference between quantitative genetic interaction scores derived from yeast grown on standard versus DNA-damaging media [6]. We found substantial changes in interaction patterns and demonstrated that the difference in scores was more effective than the scores in either static condition for highlighting interactions relevant to the pathway under study (DNA damage response (DDR)). Other biological networks, such as protein-protein interaction (PPI) or protein-DNA interaction networks, have also progressed from observing single experimental conditions to comparing the changes in interactions across multiple experimental conditions or genetic backgrounds. For example, Wrana and colleagues [9] developed the LUMIER (luminescence-based mammalian interactome mapping) strategy to identify pairwise PPIs among a set of human factors with and without stimulation by transforming growth factor β. Similarly, Workman *et al*. [10] used genome-wide chromatin immunoprecipitation to focus on changes in transcription factor binding after exposure to the DNA damaging agent methyl methanesulfonate (MMS). More recently, a quantitative approach has been presented by Bisson *et al*. [11] for measuring differential interactions in PPI networks. This approach, which the authors call affinity purification-selected reaction monitoring (AP-SRM), was used to map quantitative changes in interaction with the protein Grb2, which showed that the composition of Grb2 complexes was remarkably dependent on the stimulation. By focusing on additional hub proteins beyond Grb2, this method is likely to be useful for obtaining a global overview of protein network remodeling in response to a stimulus.

The progression from static to differential network biology in many fields increases the need for specialized statistical strategies for scoring differential networks. One approach to improving differential signal is to use paired experimental designs that reduce the noise between treated and untreated measurements. For example, experimental designs such as the two-color microarray were originally developed to reduce the noise resulting from technical variability, and various statistical methods have been developed to leverage the paired structure of these experiments (reviewed in [12-15]). Similar to two-color microarrays, differential network measurements can pair treated and untreated measurements. While some of the differential interaction studies [6,7] have employed such an experimental design, they did not utilize this information in their analysis, treating each measurement as independent.

Here, we investigate the statistical structure of two large-scale differential genetic interaction experiments [6,7] and present a generalized strategy for scoring differential genetic interaction data. Our strategy produces differential genetic interaction networks that are more reproducible and more enriched for biologically relevant interactions than previous approaches based on network subtraction. A MATLAB implementation of our strategy is provided as Additional file 1 with the online version of this article.

## Results and discussion

### The differential interaction model

**Figure 1:**
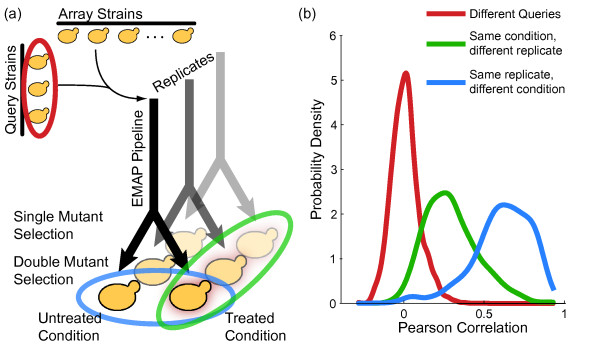
**The paired experimental pipeline**. **(a) **The pipeline for generating differential genetic interactions is the same as for static genetic interactions except for a split onto treated and untreated plates in the last step. **(b) **Normalized colony size profiles for the same experimental replicate across the two conditions (blue) have the greatest Pearson correlation, as compared to the profiles of two experimental replicates of the same condition (green) or the profiles of different queries (red). EMAP, Epistasis MAPping.

The format of a differential genetic interaction experiment takes growth-rate measurements for each double mutant across two or more conditions. A single mutant yeast strain, called the 'query', is mated with an entire set of other single mutants (for example, deletions of all non-essential yeast genes), referred to as 'array' strains. The resulting diploids are sporulated and then undergo multiple selection steps to produce colonies of haploid double deletion mutants. In the last step of the pipeline, the same yeast colonies are replicated onto different media exhibiting the chosen growth conditions (Figure 1a; see [3,6,16] for high-throughput genetic interaction screening protocols).

Because one run of this experimental pipeline produces double mutant colonies that are grown in separate conditions but share the same initial steps, we had reason to believe that the double mutant growth-rate measurements are not independent. Using data from Bandyopadhyay *et al*. [6], we tested this hypothesis by comparing the correlation of experimental replicates (that is, colonies generated in separate pipelines but grown in the same condition) with the correlation of colonies generated in the same pipeline but grown in different final conditions. Strikingly, we found that the correlation of colonies grown in different conditions was much greater than the correlation of experimental replicates (Figure 1b), even though the experimental replicates were grown under identical growth conditions and the conditional replicates were not. This observation suggested some degree of statistical dependence between the conditional replicate measurements.

We further assessed the dependence across the conditional measurements with an analysis of the variance of replicate measurements. Assuming independence, the difference between two normally distributed random variables is distributed normally, with a variance equal to the sum of the variances of the original distributions (Equation 1):

(1) $$N\left(\mu_{1},\sigma_{1}^{2}\right)-N\left(\mu_{2},\sigma_{2}^{2}\right)\sim N\left(\mu_{1}-\mu_{2},\sigma_{1}^{2}+\sigma_{2}^{2}\right)$$

Therefore, for each double mutant, the variance of the differences between the static measurements should be equal to the sum of the variances of the static measurements.

**Figure 2:**
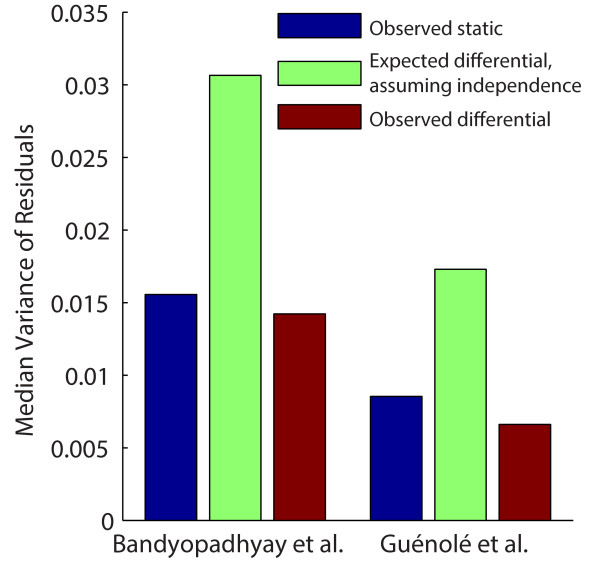
**Theoretical and observed differential variances**. Bar plot of the observed static, expected differential (assuming independence), and observed differential variances of normalized colony size residuals. The median values across all double mutants are shown. Bandyopadhyay *et al*. [6]; Guénolé et al. [7].

Using the data from two differential interaction mapping experiments comparing MMS and standard growth conditions [6,7], we found that the variance of the difference for each double mutant was less than half of the expected differential variance, and even less than the variance of static (non-differential) measurements (Figure 2). These results confirm that the across-condition measurements are not independent and raise the possibility that significant error reduction may be achieved by the differential mode of analysis.

### The dS score: a quantitative measure of differential interaction

Accordingly, we developed a strategy for scoring differential genetic interactions, which accounts for the dependency structure of the data. Assuming a growth constant *p *for each plate, which captures plate-to-plate differences in growth rate, the observed double mutant colony size *z_qai _*can be factored as follows:

(2) $$z_{qaic}=p_{qic}\cdot f_{qc}\cdot f_{ac}+\in_{qaic}$$

where *q *and *a *represent the query and array strains, *i *represents the experimental replicate, *c *represents the condition, *f *indicates the single mutant fitnesses, and*∈ *represents the residual. Collins *et al*. [17] developed a strategy that uses colony size population trends to estimate *p*, *f_q_*, and *f_a _*and obtain a measurement of the residual, which serves to quantify the degree of genetic interaction between the query and array mutants.

For differential interactions, the null or 'non-interaction' model is that the mean of the differences between paired residuals is equal to zero:

(3) $$\overset{n}{\underset{i}{\sum }}\frac{\in_{qaic}-\in_{qaic_{0}}}{n}=\overset{n}{\underset{i}{\sum }}\frac{\delta_{qaic}}{n}=0$$

where *c *indicates the treatment and *c_0 _*indicates the untreated, or reference, condition, and δ represents the difference in colony size residuals. Assuming thes*∈ *are normally distributed, the degree to which this mean differs from zero given the variance of the replicates can be modeled using the paired t-statistic. We call our statistic the dS score, 'd' for 'differential' and 'S score' after the name of the statistic used by Collins *et al*. [17]:

(4) $$\text{dS}\;\text{score = }\frac{{\bar{\delta }}_{qac}}{s_{qac}/\sqrt{n}}$$

where *δ_qac _*is the mean of the differences of the residuals (Equation 3) and *s_qac _*is the sample standard deviation of the differences of the residuals. Unlike the S-score [17], we found that the sample variance was the best approximation of the variance (based on the quality control metrics described below) and did not employ a minimum bound or any modifiers or priors (such as in the case of SAM, Cyber-T, or LIMMA in microarray analysis [15,18,19]; see also [20]).

### Similarity of differential interaction profiles provides distinct functional information

Previously, it has been shown that the correlation of static interaction profiles identifies many gene functional relationships not identified by direct genetic interactions (a genetic interaction profile is the set of all interactions with a given gene) [1,17]. Given our new quantitative score for differential interactions, we therefore investigated whether differential interaction profiles could also be used to provide distinct functional information. Indeed, we found that the correlation of differential interaction profiles was able to identify relationships relevant to the treatment response and, furthermore, that these links were not identified either by direct interactions (static or differential) or by correlation of static profiles.

**Figure 3:**
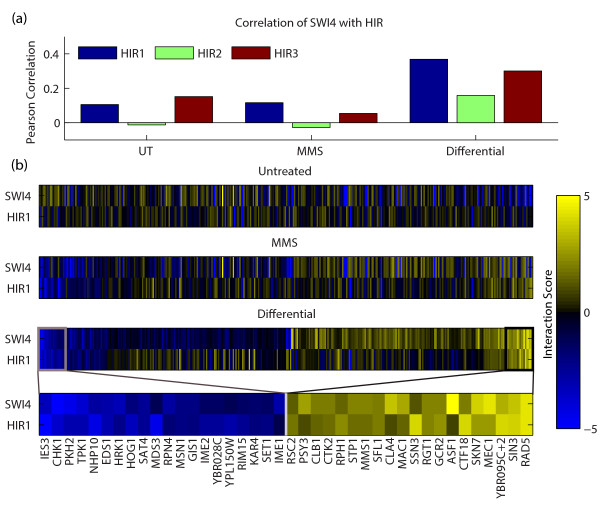
**Differential profile similarity between SWI4 and HIR**. **(a) **Bar plot showing the Pearson correlation of HIR1/2/3 profiles with SWI4 for untreated (UT), MMS, and differential (dS) scores. **(b) **Heatmaps of the untreated, MMS, and differential interaction profiles of SWI4 and HIR1; the bottom panel illustrates the interactions with greatest similarity between SWI4 and HIR1.

For example, using the dS score, we observed a very high differential similarity score between SWI4 and the subunits of the HIR complex (Figure 3). In contrast, when computing genetic profile similarity between SWI4 and HIR in either static condition (standard or MMS-treated), similarity scores were strikingly low. SWI4 is the DNA-binding member of the SBF complex, a key regulator of genes involved in DNA synthesis and repair in G1 to S phase [21,22]. HIR1, HIR2, and HIR3 are subunits of the HIR complex that negatively regulate histone protein transcription [23] under control of the DNA-damage checkpoint kinase DUN1 [24]. Although SWI4 and HIR have not been previously implicated in a genetic relationship, SWI4 has been shown to regulate histone gene expression [25,26], suggesting that an interaction between SWI4 and HIR is feasible, especially in context of the DDR. Thus, differential similarity can identify functional relationships between genes that are not apparent from profile similarity analysis in static conditions.

We identified a total of 99 functional associations like SWI4 and HIR, that is, gene pairs with low static similarity and high differential similarity (see Additional file 2 for a complete list of gene pairs and their interaction and similarity scores). These gene pairs indicate DDR-relevant interactions that would not be identified through previously available methods. One of the key limitations of static profile similarity is that the static profile is populated by interactions pertaining to both the treatment as well as general cell growth. These non-relevant interactions diminish the similarity between genes that otherwise function very similarly in the treatment response. Additionally, the larger variance inherent in the static measurements contributes to noisier interaction profiles, which decreases the similarity of otherwise related profiles. Differential interactions are effective at identifying treatment-relevant relationships because they cut down the noise and eliminate non-related interactions.

### Performance of the dS score and differential profile similarity

**Figure 4:**
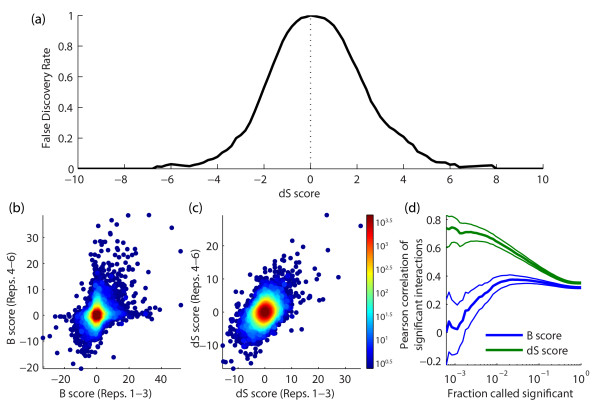
**False discovery rate and reproducibility of the dS score**. **(a) **Plot of the false discovery rate of the dS score as a function of score magnitude. **(b,c) **Scatter of differential scores calculated on independent replicate subsets using (b) the B scores and (c) the dS score; the points shown in either panel are only those scored by both analyses. **(d) **Plot comparing the Pearson correlation of significant interactions for the B and dS scores (blue and green, respectively) over a full range of significance thresholds - that is, the correlation of the top *n *percent of the interactions for n = 0.1% (left side) to n = 100% (right side); error bars (non-bolded lines) indicate the 95% confidence intervals of the correlation coefficient.

We investigated the quality of the dS score by examining its false discovery rate, reproducibility and biological enrichment. As a baseline for comparison, where applicable dS scores were compared to the differential *P*-values described by Bandyopadhyay *et al*. [6], which indicate an empirically determined significance for the difference in S scores between two conditions. We designate the -log *P*-values from Bandyopadhyay *et al*. [6] as the 'B score'. To estimate the false discovery rate of different dS score thresholds, we first generated a dS null distribution using the data from Bandyopadhyay *et al*. [6], in which the final step involved pinning each double mutant twice in the same condition. These two colonies were paired and scored as if they were colonies grown in separate conditions (corresponding to *z_qaic _*and *z_qaic0 _*in Equation 2 above). We observed that the dS score has approximately symmetric false discovery rates for positive and negative scores (Figure 4a).

Next, we assessed reproducibility of the dS score by comparing B and dS scores generated using replicates 1 to 3 and, separately, 4 to 6 from Guénolé *et al*. [7]. Using only gene pairs that were scored in both analyses, we found that the dS score yields a much tighter reproducibility across replicates than the B score (Figure 4b-c; Figure S1 in Additional file 3). In particular, the Pearson correlation across replicates was remarkably higher for the dS score than the B score (Figure 4d; the values on the far right correspond to data shown in Figure 4b,c). We found it of particular interest that for the most significant interactions, the dS score tends to greater and greater reproducibility, while the reproducibility of the B score drops to zero, indicating that for larger and larger values, the B score picks up on less and less signal.

**Figure 5:**
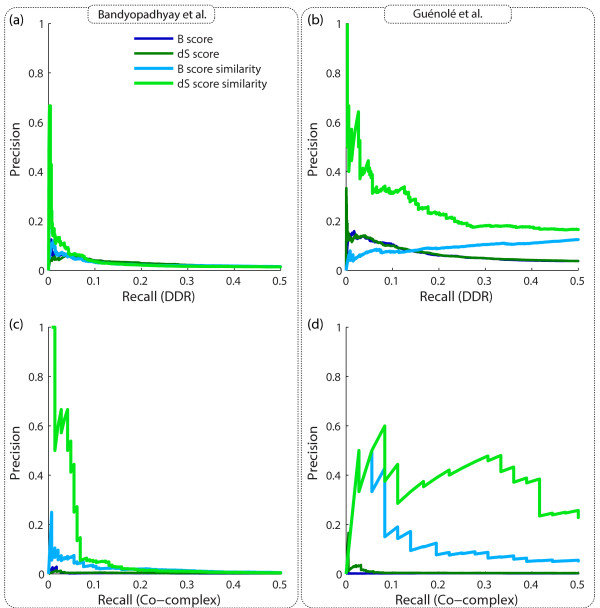
**Performance of dS score and differential profile similarity**. **(a-d) **Precision-recall plots comparing the biological enrichment of B and dS scores and their corresponding profile similarity scores for DDR interactions (a,c) and co-complex interactions (b,d) using the data from Bandyopadhyay *et al*. [6] (a,b) and Guénolé *et al*. [7] (c,d).

To measure the biological enrichment of the dS score, we generated a bronze-standard set of interactions similar to that used by Bandyopadhyay *et al*. [6]. We included in our standard set any gene pair in which both genes were annotated as 'DNA-damage response' (DDR) in the Gene Ontology [27] (corresponding to 903 or 2,575 gene pairs in the Bandyopadhyay *et al*. [6] or Guénolé *et al*. [7] data sets, respectively), as well as any gene pair defined by the YeastNet 2.0 benchmark set [28] containing at least one DDR gene (390 or 772 gene pairs, respectively). As a second standard, we used the set of co-complex interactions compiled by Baryshnikova *et al*. [29], which is based on the set of macromolecular complexes recorded in the *Saccharomyces *Genome Database [30] or in the CYC2008 protein complex catalogue [31]. Using these two standards, we generated precision-recall plots for two previously published differential interaction networks (Bandyopadhyay *et al*. [6] and Guénolé *et al*. [7]). This analysis indicated that the dS score has essentially the same precision for recovering the DDR and the co-complex standards as the original *P*-values published by Bandyopadhyay *et al*. [6] (Figure 5; see also Figures S2 to S4 in Additional file 3). However, we observed a notable improvement in enrichment for DDR interactions when using profile similarity of dS scores compared to profile similarity of B scores (Figure 5a,b).

Additionally, it is well known that gene pairs with high profile similarity are often members of the same physical complexes [32,33], so we investigated whether the same is true for differential-profile similarity. We found that the genes with similar dS score profiles are strikingly more enriched for co-complex pairs (Figure 5c,d), and specifically for protein complexes involved in the DDR (Figure S2 in Additional file 3). For example, differential profile similarity was able to achieve a precision of 60 to 100% for recovering either DDR pathway interactions or protein complexes, using data from either of two studies. This performance was in contrast to that of individual differential interactions, which had a precision of 1 to 20% using these same standards and data.

It is interesting that B score profile similarity is under-enriched for meaningful relationships. Part of this behavior may be explained by our observation that extreme B score values tend to capture noise and are not reproducible (Figure 4b-d). Because profile similarity is heavily influenced by larger values, B score profile similarity is overly sensitive to noise. Thus, relatively few spurious interactions can have an extensive influence on profile similarity.

We finally compared dS scores and dS profile similarity scores to the static S scores and profile similarity scores from the same data. We found that differential similarity scores are more enriched for DDR interactions than static similarity scores, even though static scores are more enriched for non-DDR-specific interactions (Figure S3 in Additional file 3).

The reasons for the improved performance in identifying relevant genetic relationships of the dS score over the B score and the static scores deserve some attention. Genetic interaction mapping experiments are subject to many systematic sources of noise. For example, the ratio of double mutant cells to single mutant cells in the colonies growing on the single-mutant selection plate (see Figure 1 for an outline of the experimental workflow) affects the observed double mutant fitness in the following step. Other sources of systematic noise include uneven agar surfaces, which affect the quantity of material that is picked up and deposited during plate pinning, and variations in incubation time, humidity, and so on (Table 1). Despite sophisticated data processing methods, traces of these systematic artifacts may be preserved, and this noise can influence the estimation of interaction effects. The current experimental design for static interaction mapping experiments does not control for these artifacts, and the previous method for scoring differential interactions did not take advantage of built-in controls. However, our approach uses the paired relationships between plates to eliminate many sources of systematic noise, increasing our ability to identify reproducible and relevant differential interactions (Figures 1, 2, and 4). This result is of broad interest because finding the appropriate control plays an important part in differential experimental design in many fields.

**Table 1:** Sources of noise and their effect on interaction scores

	Noise affects score?	
	
Source of noise	Static score	dS score
Double/single mutant ratio, pre-DM selection	ਲ	
Double/single mutant ratio, DM selection	ਲ	ਲ
Uneven agar surface, pre-DM selection	ਲ	
Uneven agar surface, DM selection	ਲ	ਲ
Variation in environment, pre-DM selection	ਲ	
Variation in environment, DM selection	ਲ	ਲ

DM, Double Mutant

### Interpretation of the dS score

The previous approach to scoring differential interactions derived a score from the difference between static interaction scores in each condition. This explicit comparison of scores led to a natural discussion about the interpretation of the differential score based on the sign and magnitudes of the static scores [6]. However, because the dS score is not based on the difference between static scores, we suggest the dS score be interpreted following the same logic as static interaction scores. In the static case, positive interactions generally denote gene relationships within the same pathway or complex, while negative interactions generally indicate gene relationships that span parallel or redundant pathways [34]. The difference between differential and static interpretation is that static scores indicate interactions that affect general cell growth, whereas differential scores indicate interactions that affect the treatment response.

While the theoretical interpretation of the dS score is straightforward, the practical interpretation is more complex because the static interaction scores provide a context for the interpretation of the dS score. For example, a gene pair exhibiting a positive interaction in untreated conditions that is more positive in MMS (yielding a positive dS score) should be interpreted differently than an interaction that is negative in untreated conditions that becomes positive in MMS (also yielding a positive dS score). According to the standard interaction model, the latter example is supposedly going from a between-pathway relationship in untreated conditions to a within-pathway relationship in the treatment, which quality the former example does not have, even though both examples exhibit a co-pathway relationship in the DDR response. These various classes of differential interactions exhibit different enrichment rates for our DDR standard (Figure S4 in Additional file 3), suggesting that there may be unique qualities to each class, but a more detailed investigation of differential interaction interpretation is left for future work.

## Conclusions

Here, we have put forth a quantitative differential interaction score, the dS score, based on important statistical information inherent in the experimental design. This score not only provides more information about each interaction than previous approaches, but also shows improved reproducibility and comparable biological enrichment. Additionally, quantitative differential interactions give rise to differential interaction profiles, which we demonstrate to be biologically relevant and uniquely insightful. Furthermore, we provide a new interpretation for differential interactions based on the accepted interpretation of static genetic interactions. We conclude that our differential interaction score is preferred to the previous approach for differential genetic interaction mapping analysis.

## Materials and methods

### Correlation of query replicates

We used normalized colony size residuals to calculate the correlation of query replicates (Figure 1b). Our approach to computing these residuals is based on the approach published by Collins *et al*. [17]. In brief, the raw colony sizes are pre-processed to filter bad colonies and correct spatial artifacts. Each plate (that is, the set of all colony sizes from the same plate) is normalized by the plate mode, calculated using a kernel density estimation method [35]. Next, array single mutant fitnesses are estimated using the median normalized colony size for a given array position across all plates, which are then subtracted from the respective double mutant colony sizes to yield normalized colony size residuals. These residuals are, in turn, used to calculate several quantities: (1) the pair-wise correlation for each pair of conditional plate replicates, that is, double mutant selection plates derived from the same single mutant selection plate differing only in the growth condition; (2) pairwise correlation of untreated experimental replicates; and (3) pairwise correlation of randomly selected queries.

### The dS score

Normalized differentials are obtained by subtracting untreated normalized colony sizes from the corresponding treated normalized colony sizes. The dS score is then computed as the pooled t-statistic of the six replicates for a given double mutant versus all double mutant measurements containing the respective array gene deletion. Note that the S score, for scoring static interactions, employs a minimum bound on the variance of the six double mutant replicates [17], while the dS score does not bound the variance.

### Scoring null differential interactions

The null distribution of dS scores was generated by using replicate pairs of measurements grown on the same plate (and therefore same condition) and following the same scoring procedure already described. The differentials for the three replicates in each condition were pooled to produce six total replicates for each gene pair. We computed false discovery rates for each dS score cutoff as the ratio of the proportion of null scores beyond the cutoff to the proportion of observed dS scores beyond the cutoff.

### Biological enrichment

The 'bronze' standard for differential genetic interactions in response to DNA damage was compiled as (1) the set of all gene pairs in which both genes are annotated as 'DNA damage response' (DDR) in the Gene Ontology [27] (term ID GO:0006974, direct association; accessed December 2011), and (2) the set of all gene pairs indicated by the YeastNet 2.0 benchmark set [28] in which at least one gene is annotated as DDR. The lists of DDR genes and bronze-standard DDR gene pairs are provided as Additional file 4.

The gold standard used for co-complex membership is defined by Baryshnikova *et al*. [29]. Precision-recall plots were computed using the absolute value of the dS scores (treating positive and negative interactions equally).

### Significance of Pearson correlation

To assess the significance of the difference between the correlation coefficients of the scores in Figure 3, we calculated the correlation of bootstrapped data for 10,000 iterations in a paired fashion and counted the number of cases in which the correlation of B scores was greater than the correlation of the dS scores.

### Determining associations similar to SWI4-HIR

To identify gene associations similar to SWI4 and HIR, where the differential similarity is high and the static similarity is low, we used the cutoffs of >0.35 and <0.15 for differential and static similarity scores, respectively.

## Abbreviations

DDR, DNA-damage response; MMS, methyl methanesulfonate; PPI, protein-protein interaction.

## Competing interests

The authors declare that they have no competing interests.

## Authors' contributions

GB developed the statistical model and performed the validation and discovery. Both authors read and approved the final manuscript.

## Supplementary Material

Additional file 1 **MATLAB implementation of our method**. Click here for file

Additional file 2 **A table of the dS, S, and profile similarity scores for the data from Bandyopadhyay *et al***. [6]. Click here for file

Additional file 3 **a PDF containing our additional notes and figures**. Click here for file

Additional file 4 **a table indicating the gene pairs used as the DNA damage response bronze standard in our study**. Click here for file

## References

[B1] Costanzo M Baryshnikova A Bellay J Kim Y Spear ED Sevier CS Ding H Koh JLY Toufighi K Mostafavi S Prinz J St Onge RP VanderSluis B Makhnevych T Vizeacoumar FJ Alizadeh S Bahr S Brost RL Chen Y Cokol M Deshpande R Li Z Lin Z-Y Liang W Marback M Paw J San Luis B-J Shuteriqi E Tong AHY van Dyk N The genetic landscape of a cell. Science 2010 13 425 431 10.1126/science.1180823 20093466 PMC5600254

[B2] Fiedler D Braberg H Mehta M Chechik G Cagney G Mukherjee P Silva AC Shales M Collins SR van Wageningen S Kemmeren P Holstege FCP Weissman JS Keogh M-C Koller D Shokat KM Krogan NJ Functional organization of the S. cerevisiae phosphorylation network. Cell 2009 13 952 963 10.1016/j.cell.2008.12.039 19269370 PMC2856666

[B3] Schuldiner M Collins SR Thompson NJ Denic V Bhamidipati A Punna T Ihmels J Andrews B Boone C Greenblatt JF Weissman JS Krogan NJ Exploration of the function and organization of the yeast early secretory pathway through an epistatic miniarray profile. Cell 2005 13 507 519 10.1016/j.cell.2005.08.031 16269340

[B4] Collins SR Miller KM Maas NL Roguev A Fillingham J Chu CS Schuldiner M Gebbia M Recht J Shales M Ding H Xu H Han J Ingvarsdottir K Cheng B Andrews B Boone C Berger SL Hieter P Zhang Z Brown GW Ingles CJ Emili A Allis CD Toczyski DP Weissman JS Greenblatt JF Krogan NJ Functional dissection of protein complexes involved in yeast chromosome biology using a genetic interaction map. Nature 2007 13 806 810 10.1038/nature05649 17314980

[B5] Zheng J Benschop JJ Shales M Kemmeren P Greenblatt J Cagney G Holstege F Li H Krogan NJ Epistatic relationships reveal the functional organization of yeast transcription factors. Mol Systems Biol 2010 13 420 10.1038/msb.2010.77 PMC2990640 20959818

[B6] Bandyopadhyay S Mehta M Kuo D Sung M-K Chuang R Jaehnig EJ Bodenmiller B Licon K Copeland W Shales M Fiedler D Dutkowski J Guénolé A van Attikum H Shokat KM Kolodner RD Huh W-K Aebersold R Keogh M-C Krogan NJ Ideker T Rewiring of genetic networks in response to DNA damage. Science 2010 13 1385 1389 10.1126/science.1195618 21127252 PMC3006187

[B7] Guénolé A Srivas R Vreeken K Wang S Krogan NJ Ideker T van Attikum H Dissection of DNA damage response pathways using a multi-conditional genetic interaction map. Mol Cell 2012 13 346 358 23273983 10.1016/j.molcel.2012.11.023 PMC3633480

[B8] Ideker T Krogan NJ Differential network biology. Mol Systems Biol 2012 13 1 9 10.1038/msb.2011.99 PMC3296360 22252388

[B9] Barrios-Rodiles M Brown KR Ozdamar B Bose R Liu Z Donovan RS Shinjo F Liu Y Dembowy J Taylor IW Luga V Przulj N Robinson M Suzuki H Hayashizaki Y Jurisica I Wrana JL High-throughput mapping of a dynamic signaling network in mammalian cells. Science 2005 13 1621 1625 10.1126/science.1105776 15761153

[B10] Workman CT Mak HC McCuine S Tagne J-B Agarwal M Ozier O Begley TJ Samson LD Ideker T A systems approach to mapping DNA damage response pathways. Science 2006 13 1054 1059 10.1126/science.1122088 16709784 PMC2811083

[B11] Bisson N James DA Ivosev G Tate S a Bonner R Taylor L Pawson T Selected reaction monitoring mass spectrometry reveals the dynamics of signaling through the GRB2 adaptor Nat Biotechnol 2011 13 653 658 10.1038/nbt.1905 21706016

[B12] Patterson T a Lobenhofer EK Fulmer-Smentek SB Collins PJ Chu T-M Bao W Fang H Kawasaki ES Hager J Tikhonova IR Walker SJ Zhang L Hurban P de Longueville F Fuscoe JC Tong W Shi L Wolfinger RD Performance comparison of one-color and two-color platforms within the MicroArray Quality Control (MAQC) project. Nat Biotechnol 2006 13 1140 1150 10.1038/nbt1242 16964228

[B13] Cui X Churchill G a Statistical tests for differential expression in cDNA microarray experiments Genome Biol 2003 13 210 10.1186/gb-2003-4-4-210 12702200 PMC154570

[B14] Cui X Hwang JTG Qiu J Blades NJ Churchill G a Improved statistical tests for differential gene expression by shrinking variance components estimates Biostatistics (Oxford, England) 2005 13 59 75 10.1093/biostatistics/kxh018 15618528

[B15] Smyth GK Linear models and empirical bayes methods for assessing differential expression in microarray experiments Stati Appl Genet Mol Biol 2004 13 Article3 10.2202/1544-6115.1027 16646809

[B16] Tong a H Evangelista M Parsons a B Xu H Bader GD Pagé N Robinson M Raghibizadeh S Hogue CW Bussey H Andrews B Tyers M Boone C Systematic genetic analysis with ordered arrays of yeast deletion mutants. Science 2001 13 2364 2368 10.1126/science.1065810 11743205

[B17] Collins SR Schuldiner M Krogan NJ Weissman JS A strategy for extracting and analyzing large-scale quantitative epistatic interaction data Genome Biol 2006 13 R63 10.1186/gb-2006-7-7-r63 16859555 PMC1779568

[B18] Tusher VG Tibshirani R Chu G Significance analysis of microarrays applied to the ionizing radiation response Proc Natl Acad Sci USA 2001 13 5116 5121 10.1073/pnas.091062498 11309499 PMC33173

[B19] Baldi P Long AD A Bayesian framework for the analysis of microarray expression data: regularized t-test and statistical inferences of gene changes Bioinformatics 2001 13 509 519 10.1093/bioinformatics/17.6.509 11395427

[B20] Murie C Woody O Lee AY Nadon R Comparison of small n statistical tests of differential expression applied to microarrays BMC Bioinformatics 2009 13 45 10.1186/1471-2105-10-45 19192265 PMC2674054

[B21] Sidorova J Breeden L Analysis of the SWI4/SWI6 protein complex, which directs G1/S-specific transcription in Saccharomyces cerevisiae Mol Cell Biol 1993 13 1069 1077 8423776 10.1128/mcb.13.2.1069-1077.1993 PMC358992

[B22] Ho Y Mason S Kobayashi R Hoekstra M Andrews B Role of the casein kinase I isoform, Hrr25, and the cell cycle-regulatory transcription factor, SBF, in the transcriptional response to DNA damage in Saccharomyces cerevisiae Proc Natl Acad Sci USA 1997 13 581 586 10.1073/pnas.94.2.581 9012827 PMC19556

[B23] Spector M Raff A DeSilva H Hir1p and Hir2p function as transcriptional corepressors to regulate histone gene transcription in the Saccharomyces cerevisiae cell cycle. Mol Cell Biol 1997 13 545 552 9001207 10.1128/mcb.17.2.545 PMC231779

[B24] Sharp J a Rizki G Kaufman PD Regulation of histone deposition proteins Asf1/Hir1 by multiple DNA damage checkpoint kinases in Saccharomyces cerevisiae Genetics 2005 13 885 899 10.1534/genetics.105.044719 16020781 PMC1456847

[B25] Kato M Hata N Banerjee N Futcher B Zhang MQ Identifying combinatorial regulation of transcription factors and binding motifs Genome Biol 2004 13 R56 10.1186/gb-2004-5-8-r56 15287978 PMC507881

[B26] Eriksson PR Ganguli D Clark DJ Spt10 and Swi4 control the timing of histone H2A/H2B gene activation in budding yeast Mol Cell Biol 2011 13 557 572 10.1128/MCB.00909-10 21115727 PMC3028627

[B27] Ashburner M Ball CA Blake JA Botstein D Butler H Cherry JM Davis AP Dolinski K Dwight SS Eppig JT Harris MA Hill DP Issel-Tarver L Kasarskis A Lewis S Matese JC Richardson JE Ringwald M Rubin GM Sherlock G Gene ontology: tool for the unification of biology. The Gene Ontology Consortium. Nat Genet 2000 13 25 29 10.1038/75556 10802651 PMC3037419

[B28] Lee I Li Z Marcotte EM An improved, bias-reduced probabilistic functional gene network of baker's yeast, Saccharomyces cerevisiae PloS One 2007 13 e988 10.1371/journal.pone.0000988 17912365 PMC1991590

[B29] Baryshnikova A Costanzo M Kim Y Ding H Koh J Toufighi K Youn J-Y Ou J San Luis B-J Bandyopadhyay S Hibbs M Hess D Gingras A-C Bader GD Troyanskaya OG Brown GW Andrews B Boone C Myers CL Quantitative analysis of fitness and genetic interactions in yeast on a genome scale. Nat Methods 2010 13 1017 1024 10.1038/nmeth.1534 21076421 PMC3117325

[B30] *Saccharomyces *Genome Database. http://www.yeastgenome.org/

[B31] Pu S Wong J Turner B Cho E Wodak SJ Up-to-date catalogues of yeast protein complexes. Nucleic Acids Res 2009 13 825 831 10.1093/nar/gkn1005 19095691 PMC2647312

[B32] Srivas R Hannum G Ruscheinski J Ono K Wang P-L Smoot M Ideker T Assembling global maps of cellular function through integrative analysis of physical and genetic networks Nat Protocols 2011 13 1308 1323 10.1038/nprot.2011.368 PMC3383003 21886098

[B33] Bandyopadhyay S Kelley R Krogan NJ Ideker T Functional maps of protein complexes from quantitative genetic interaction data. PLoS Comput Biol 2008 13 e1000065 10.1371/journal.pcbi.1000065 18421374 PMC2289880

[B34] Boone C Bussey H Andrews BJ Exploring genetic interactions and networks with yeast. Nat Rev Genet 2007 13 437 449 10.1038/nrg2085 17510664

[B35] Parzen E On estimation of a probability density function and mode Ann Mathematical Stat 1962 13 1065 1076 10.1214/aoms/1177704472

