# Supplementary material for: Differential analysis of high-throughput quantitative genetic interaction data
Source: Genome Biol. 2012 Dec 26;13(12):R123. doi: 10.1186/gb-2012-13-12-r123 (PMC4056373; doi:10.1186/gb-2012-13-12-r123)
Supplement: Additional file 3 — a PDF containing our additional notes and figures. [file gb-2012-13-12-r123-S3.PDF]

Additional Notes and Figures

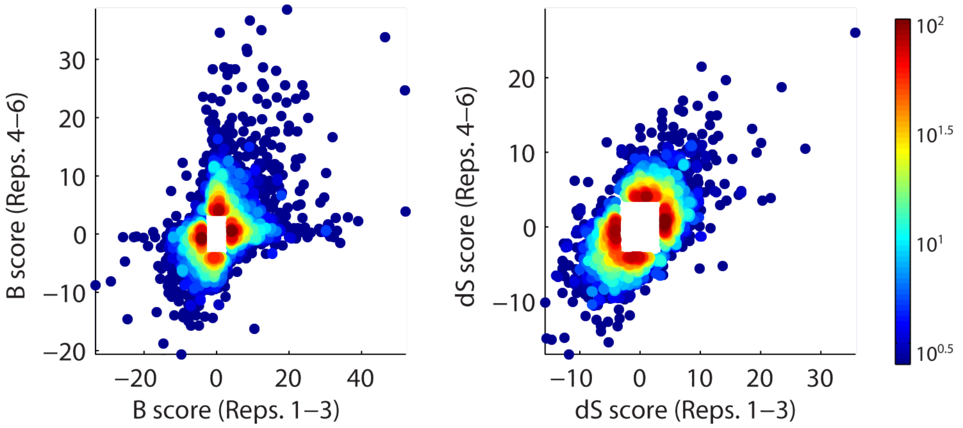

Figure S1 - dS score reproducibility, with cutoff. Scatter plots showing the reproducibility of dS scores (see Figure 4 in the main text), but showing only the top 1000 interactions in each set. With this cutoff, the B score has a Pearson correlation coefficient of 0.37 while the dS score has a Pearson correlation coefficient of 0.60.

Figure S2 - Differential enrichment for DDR complexes. Histogram of dS profile similarity scores (Pearson correlation) for DDR Co-complex, co-complex, and other interactions. Gene pairs belonging to the same DDR complexes tend to have profiles that are correlated.

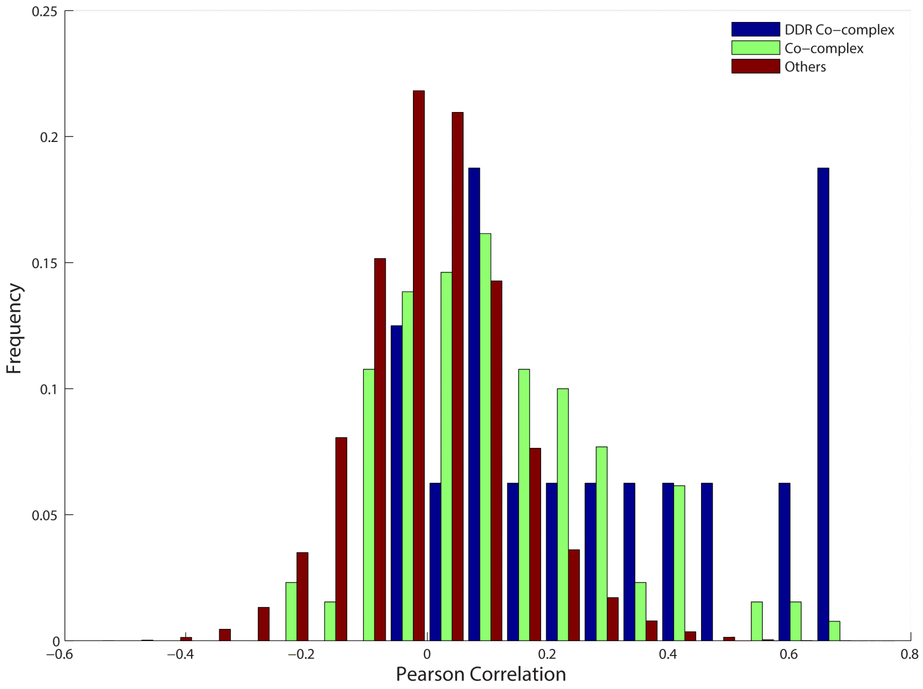

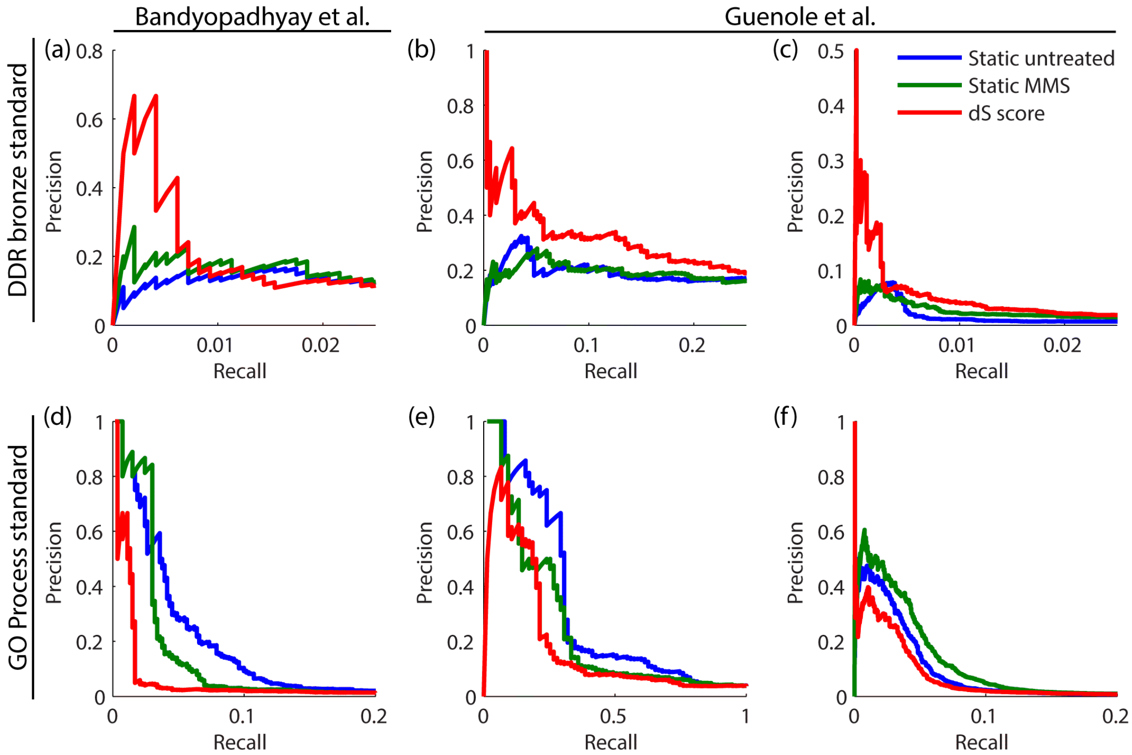

Figure S3 - Differential versus static profile similarity. (a) Precision-recall plot indicating the enrichment of static untreated, static MMS, and differential (dS score) profile similarity for DDR interactions using the data from Bandyopadhyay et al. **(b-c)** Precision-recall plots indicating the enrichment of static untreated, static MMS, and differential query (b) and array (c) profile similarity for DDR interactions using the data from Guenole et al. **(d-f)** Same as (a-c), but using the GO Process co-annotation standard.

Additional Note 1

To better understand the strengths of the dS score, we evaluated the positive and negative dS score separately and found a striking trend: the positive dS scores account for nearly all of the functional enrichment (Figure S4 a-b). While such results may cause the reader to wonder if the negative dS score has any value given its low functional enrichment, we observed that negative dS scores contribute to the profile similarity of functionally related genes, indicating that while these interactions are not DDR (according to our bronze-standard), they do encode important biological information. Additionally, Bandyopadhyay et al. observed that DDR-related complexes are often linked by negative differential interactions [1]. This suggests a strong bias towards co-pathway relationships in the bronze-standard, which makes sense given the bronze-standard is derived from co-DDR genes pairs (i.e. gene pairs where both genes are annotated as DDR) and interactions defined by YeastNet [2], which maximizes for gene pairs that are functionally similar.

We further investigated the positive dS scores, breaking them up into those that are negative in untreated and become less negative in MMS (negative/negative), those that

are negative in untreated and become positive in MMS (negative/positive), and those that are positive in untreated and become more positive in MMS (positive/positive). We found that both positive/positive and negative/positive interactions achieved high precision, while negative/negative interactions enriched very little (Figure S4 c-d). Again, because our bronze-standard is heavily biased towards co-pathway relationships, negative/negative interactions, which do not suggest a co-pathway relationship in either condition, will likely not enrich for our bronze-standard.

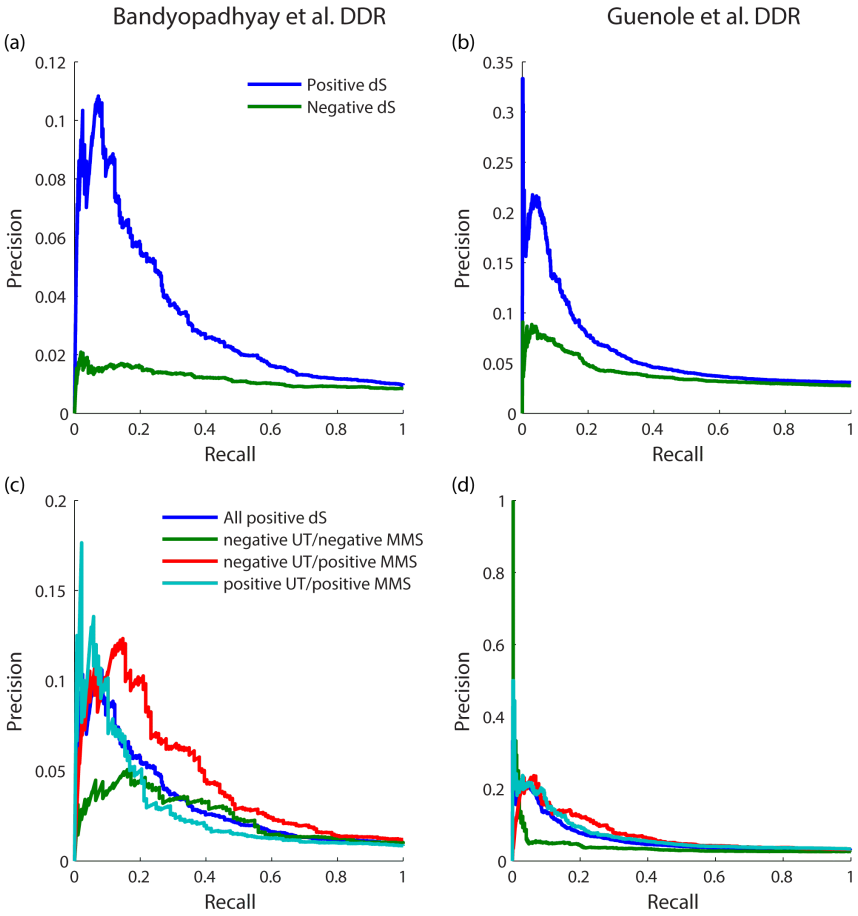

Figure S4 - Positive versus negative dS scores. (a-b) Precision-recall plots comparing the enrichment of positive and negative dS scores for DNA-damage response (DDR) interactions for data from Bandyopadhyay et al. (a) and Guenole et al. (b); **(c-d)** Precision-recall plots comparing the enrichment of positive dS scores from different static score cases for DDR interactions using data from Bandyopadhyay et al. (c) and Guenole et al. (d).

Because our original bronze-standard has such a strong bias towards co-pathway relationships, we tried to reproduce the analysis shown in Figure 5 using another bronze-standard that is less orthogonal to negative interactions. However, we found this task to be non-trivial and beyond the scope of this study. Nonetheless, we are confident that negative differential interactions encode biologically meaningful information because of their reported role in inter-complex interactions [1].

While a more thorough investigation of the merits and meaning of negative interactions is left for future work, we briefly investigated the value of negative differential interactions by examining their contribution to the correlation of similar differential profiles. We created two copies of each data set (i.e. Bandyopadhyay et al. and Guenole et al.) and replaced the negative and positive interactions with noise drawn from the distribution of negative and positive interactions, respectively. We then computed the profile similarity

of the modified profiles and compared the similarity scores of the no-positive and no-negative data sets. We found that profile similarity scores computed on these data were very similar (Figure S5), indicating that positive and negative interactions contribute very similar information to the profile similarity signal. Thus, while the exact function of negative differential interactions is unclear, they are consistent with the information provided by the positive differential interactions.

We also compared the enrichment rates of positive and negative differential interactions using a non-DDR gold standard (GO Process co-annotated gene pairs [3]) and found that while positive interactions show a greater enrichment among the genes in the study by Bandyopadhyay et al., negative interactions show a greater enrichment among genes in the Guenole et al. study (Figure S6).

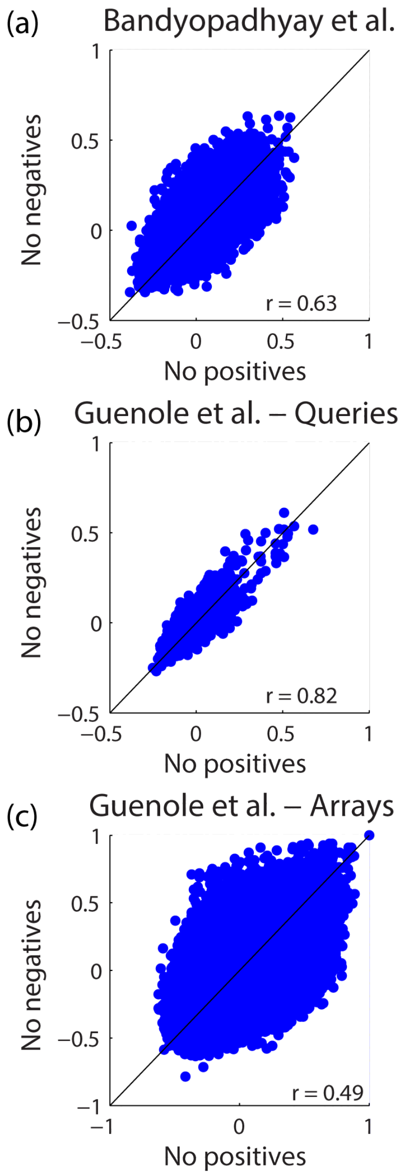

Figure S5 – Contribution of positive and negative interactions to profile similarity. (a-c) Scatter plots of the profile similarity scores based on the no-positive and no-negative copies of the Bandyopadhyay et al. (a) and Guenole et al. query (b) and array (c) interaction profiles.

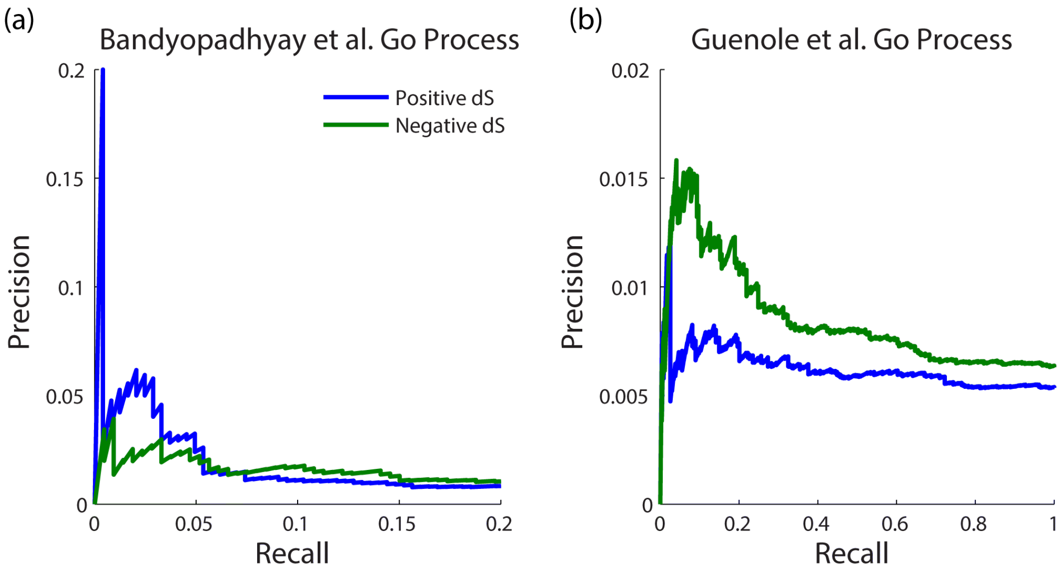

Figure S6 – Enrichment of dS scores, by class, for GO Process co-annotated gene pairs. (a-b) Precision-recall plots comparing the enrichment of positive and negative dS scores for GO Process co-annotated gene pairs for data from Bandyopadhyay et al. (a) and Guenole et al. (b).

References

1. Bandyopadhyay S, Mehta M, Kuo D, Sung M-K, Chuang R, Jaehnig EJ, Bodenmiller B, Licon K, Copeland W, Shales M, Fiedler D, Dutkowski J, Guénolé A, van Attikum H, Shokat KM, Kolodner RD, Huh W-K, Aebersold R, Keogh M-C, Krogan NJ, Ideker T: **Rewiring of genetic networks in response to DNA damage.** *Science (New York, N.Y.)* 2010, **330**:1385–910.1126/science.1195618.

2. Lee I, Li Z, Marcotte EM: **An improved, bias-reduced probabilistic functional gene network of baker's yeast, Saccharomyces cerevisiae.** *PloS one* 2007, **2**:e98810.1371/journal.pone.0000988.

3. Ashburner M, Ball CA, Blake JA, Botstein D, Butler H, Cherry JM, Davis AP, Dolinski K, Dwight SS, Eppig JT, Harris MA, Hill DP, Issel-Tarver L, Kasarskis A, Lewis S, Matese JC, Richardson JE, Ringwald M, Rubin GM, Sherlock G: **Gene ontology: tool for the unification of biology. The Gene Ontology Consortium.** *Nature genetics* 2000, **25**:25–910.1038/75556.
